# DNA methylation and aeroallergen sensitization: The chicken or the egg?

**DOI:** 10.1186/s13148-022-01332-5

**Published:** 2022-09-16

**Authors:** Anna Kilanowski, Simon Kebede Merid, Sarina Abrishamcar, Dakotah Feil, Elisabeth Thiering, Melanie Waldenberger, Erik Melén, Annette Peters, Marie Standl, Anke Hüls

**Affiliations:** 1 Institute of Epidemiology, Helmholtz Zentrum München - German Research Center for Environmental Health, Neuherberg, Germany; 2 Institute for Medical Information Processing, Biometry, and Epidemiology; Pettenkofer School of Public Health, LMU Munich, Munich, Germany; 3 Department of Epidemiology, Rollins School of Public Health, Emory University, 1518 Clifton Rd NE, Atlanta, GA 30322 USA; 4 Division of Metabolic and Nutritional Medicine, Dr. Von Hauner Children’s Hospital, University of Munich Medical Center, Munich, Germany; 5 Department of Clinical Science and Education, Södersjukhuset, Karolinska Institutet, Stockholm, Sweden; 6 Research Unit Molecular Epidemiology, Helmholtz Zentrum München - German Research Center for Environmental Health, Neuherberg, Germany; 7 Chair of Epidemiology, Ludwig-Maximilians University, Marchioninistr. 15, 81377 Munich, Germany; 8 German Center for Lung Research (DZL), Munich, Germany; 9 Gangarosa Department of Environmental Health, Rollins School of Public Health, Emory University, Atlanta, GA USA

**Keywords:** High-dimensional mediation analysis, DNA methylation, Allergic diseases, Epidemiology, Methylation risk scores, Polygenic risk scores, Maternal smoking

## Abstract

**Background:**

DNA methylation (DNAm) is considered a plausible pathway through which genetic and environmental factors may influence the development of allergies. However, causality has yet to be determined as it is unknown whether DNAm is rather a cause or consequence of allergic sensitization. Here, we investigated the direction of the observed associations between well-known environmental and genetic determinants of allergy, DNAm, and aeroallergen sensitization using a combination of high-dimensional and causal mediation analyses.

**Methods:**

Using prospectively collected data from the German LISA birth cohort from two time windows (6–10 years: *N* = 234; 10–15 years: *N* = 167), we tested whether DNAm is a cause or a consequence of aeroallergen sensitization (specific immunoglobulin E > 0.35kU/l) by conducting mediation analyses for both effect directions using maternal smoking during pregnancy, family history of allergies, and a polygenic risk score (PRS) for any allergic disease as exposure variables. We evaluated individual CpG sites (EPIC BeadChip) and allergy-related methylation risk scores (MRS) as potential mediators in the mediation analyses. We applied three high-dimensional mediation approaches (HIMA, DACT, gHMA) and validated results using causal mediation analyses. A replication of results was attempted in the Swedish BAMSE cohort.

**Results:**

Using high-dimensional methods, we identified five CpGs as mediators of prenatal exposures to sensitization with significant (adjusted *p* < 0.05) indirect effects in the causal mediation analysis (maternal smoking: two CpGs, family history: one, PRS: two). None of these CpGs could be replicated in BAMSE. The effect of family history on allergy-related MRS was significantly mediated by aeroallergen sensitization (proportions mediated: 33.7–49.6%), suggesting changes in DNAm occurred post-sensitization.

**Conclusion:**

The results indicate that DNAm may be a cause or consequence of aeroallergen sensitization depending on genomic location. Allergy-related MRS, identified as a potential cause of sensitization, can be considered as a cross-sectional biomarker of disease. Differential DNAm in individual CpGs, identified as mediators of the development of sensitization, could be used as clinical predictors of disease development.

**Supplementary Information:**

The online version contains supplementary material available at 10.1186/s13148-022-01332-5.

## Background

With the rise of available DNA methylation (DNAm) data in multiple cohort studies, the number of epigenome-wide association studies (EWAS) demonstrating a connection between DNAm and allergic diseases has increased. Over the last decade, EWAS reported associations of single CpGs (addition of a methyl-group to a cytosine in the context of CpG dinucleotides) with several allergic outcomes: High total immunoglobulin E (IgE) [1, 2], an antibody involved in Type I immune response and highly associated with allergic diseases, specific IgE [3] against certain aeroallergens and specific IgE plus skin-prick test [4] and meta-analyses on asthma [5] and any allergic disease [6]. Many of these CpGs have been successfully replicated in independent cohorts, and we could verify the robustness of these findings via replication of significant hits in the German LISA study [7].

However, it is unknown whether DNAm changes occur in response to allergic disease or if differential DNAm can serve as predictor of future development of allergies. Looking at aeroallergen sensitization, an objectively measured indicator of allergic diseases, we previously reported that methylation risk scores (MRS), which are defined as a weighted sum of methylation beta estimates, can be considered as cross-sectional biomarkers of current sensitization [7]. However, the predictive capabilities in prospective associations with aeroallergen sensitization were limited, indicating that DNAm might be a result rather than a predictor of allergic sensitization. On the other hand, studies investigating DNAm in cord-blood found associations with higher IgE levels later in life [8, 9], indicating a certain predictive potential.

One way to investigate this “chicken or egg—what came first?” question is a causal mediation analysis with data on exposure, mediator and outcome from three subsequent time points. Known determinants of allergic disease that can be used as exposures in such mediation analyses include genetic and environmental factors. Allergic diseases are highly heritable, with heritability estimates for allergic diseases being described as high as 91.7% for asthma [10], 90% for atopic dermatitis [11], 91% for allergic rhinitis and 68% for specific serum IgE (reviewed in Ober and Yao [12]). Additionally, numerous genetic variants associated with allergic diseases have been identified in multiple genome-wide association studies (GWAS), e.g., for atopic dermatitis [13], rhinitis [14] or any allergic disease [15, 16]. Polygenic risk scores (PRS) have been proposed to summarize genetic susceptibility to allergic diseases in one score for allergic trajectories [17] or asthma prediction [18, 19], presenting a significant association and a predictive area-under-the-curve of up to 0.59 for early transient asthma phenotypes and 0.58 for intermediate-onset wheeze [18]. However, as genetic variation in complex diseases represents a risk increase but not a certainty of disease onset as in monogenic diseases, family history of allergic diseases can be additionally considered as a proxy for the combination of allergic inheritance and environmental risk.

Further looking at environmental risk factors, maternal smoking during pregnancy represents a well-established environmental risk factor, which has been shown to influence allergic outcomes, especially asthma [20], and has also been biologically validated in preclinical mouse models [21].

A methodological challenge of investigating the “chicken or egg” question in causal mediation analyses is the high-dimensionality of DNAm data with up to 850K CpG sites being measured with the most recent Illumina DNAm arrays (Illumina MethylationEPIC BeadChip microarray). Several approaches have been proposed to address high-dimensionality in mediation analysis including (1) dimension-reduction methods, e.g., by using MRS, (2) integration of prior knowledge by only focusing on CpG sites with a known association with the exposure or outcome (or both) and (3) hypothesis-generating high-dimensional mediation analyses (HMA).

The objective of this study is to determine the causality of the observed associations between changes in DNAm and the development of allergen sensitization using HMA and MRS. We conduct different HMA at two subsequent time points using well-established determinants of allergic disease (maternal smoking during pregnancy, family history of allergies and a PRS for any allergic disease) as exposures and prospective measurements of DNAm and aeroallergen sensitization as mediators and outcomes.

## Methods

### Study population

For this study, we used data from a population-based German birth cohort on the Influence of **L**ife-style factors on Development of the **I**mmune **S**ystem and **A**llergies in East and West Germany (LISA). From 1997 to 1999, a total of 3,097 full-term healthy newborns were recruited at four study centers (Munich, Wesel, Leipzig and Bad Honnef). The study was approved by local ethics committees (Bavarian Board of Physicians, Board of Physicians of North-Rhine-Westphalia and Medical Faculty of the University of Leipzig) and written, informed consent was obtained from the parents or legal guardians. In the present study, only data from participants enrolled in the Munich study center with parental consent for genetic analyses at both six and ten years is included (*N*_max_ = 240).

### Aeroallergen sensitization

Positive aeroallergen sensitization was defined as a specific IgE threshold of > 0.35 kU/L (at least Radio-Allergo-Sorbent-Test (RAST) class one), measured for a mix of common aeroallergens (SX1 mix: Dermatophagoides pteronyssinus, cat, dog, rye, timothy grass, Cladosporium herbarum, birch and mugwort). Serum at six, ten and 15 years was analyzed using the CAP-RAST FEIA system (Pharmacia Diagnostics, Freiburg, Germany) according to the manufacturer’s instructions.

### Risk factors for aeroallergen sensitization

Genome-wide data in the LISA study were measured using the Affymetrix Chip 5.0 and 6.0 (Thermo Fisher Scientific, USA). More information on genetic data can be found in the supplementary material of Grosche et al. [22]. We calculated a PRS for any allergic disease based on the genome-wide significant hits reported in Ferreira et al. [15, 16]. Single nucleotide polymorphisms (SNPs) were extracted for each participant and weighted with the reported effect size. Multiallelic SNPs, highly correlated variants (Linkage disequilibrium R2 > 0.7), those with a low imputation quality (< 0.4) or a minor allele frequency of less than 1% were excluded. Further information on quality control and PRS calculation can be found elsewhere [7, 23].

Information on family history of allergic diseases was collected at birth and defined as a binary factor indicating no family history or at least one biological parent reporting ever experiencing asthma, atopic dermatitis or hay fever.

Maternal smoking during pregnancy was defined as smoking in the second and/or third trimester of pregnancy, with controls defined as either stopped smoking before the second trimester or never smoking. Potential confounders after literature research are sex, age, season at blood withdrawal, cell-type proportions, Body-Mass-Index (BMI), socio-economic status (SES), and air pollution, defined as nitrogen dioxide (NO_2_) at birth address (Additional file 2: Table S1).

### DNAm data

DNAm was measured for 256 participants from blood clots taken at six and ten years using the Methylation EPIC BeadChip (Illumina, Inc., San Diego, CA). We applied functional normalization [24] and ComBat [25] to normalize the data and remove technical variation. Probes were removed if they were located on the sex chromosomes, had missing values, or failed the detection *p* value of 0.01 in more than 1% of samples. Samples were removed if they were outliers, sex mismatches, or did not fulfill the bad-sample threshold of methylated and unmethylated intensities. Cell-type proportions were estimated using the *EpiDISH* package [26]. Further information on quality control and data processing can be found elsewhere [7].

### Methylation risk scores

MRS were calculated for six allergy-related EWAS, namely high IgE [2], aeroallergen sensitization [3], asthma [5], any allergic disease [6] and two on atopy, defined as high total IgE [1] or positive specific IgE as well as a positive skin-prick test [4]. Details on the calculation and evaluation of these allergy-related MRS have been published previously [7]. In short, we calculated each MRS by weighting the CpG beta-values with the respective effect size identified by the EWAS and transformed to z-scores. The selection of CpG sites was conducted using a pruning and thresholding approach [27]. As described previously [7], the MRS that reached the highest prediction accuracy for allergic sensitization at six years of age across all *p*-value thresholds was used in the downstream analyses.

### Statistical analysis

**Fig. 1:**
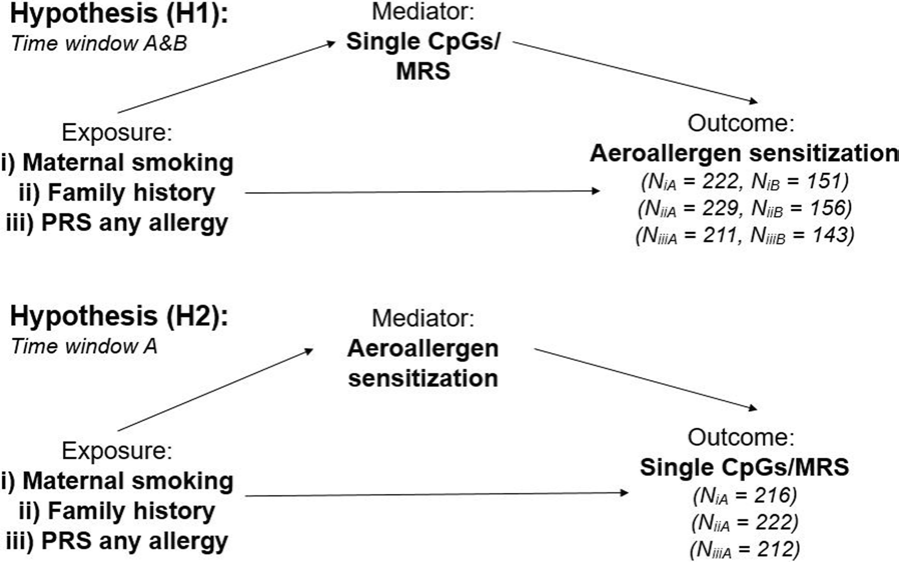
Display of models used for the identification and validation of potential mediators. Hypothesis (H1) describe the mediation of aeroallergen sensitization through DNAm and hypothesis (H2) the reversed direction that sensitization is mediating DNAm changes. Time window A covers the development from six to ten years and time window B from ten to 15 years. See also Additional file 1: Figure S1

To evaluate whether changes in DNAm are predictors or consequences of allergic diseases, we tested the following two hypotheses: (H1, DNAm as predictor) The association between exposure (maternal smoking during pregnancy; family history of allergic disease; PRS for any allergies) and allergic sensitization is mediated by prior changes in DNAm (measured by MRS or methylation in individual CpG sites); (H2, DNAm as consequence) The association between exposure (maternal smoking during pregnancy; family history of allergic disease; PRS for any allergies) and changes in DNAm (measured by MRS or methylation in individual CpG sites) is mediated by prior allergic sensitization. In our main analyses, mediators were measured at six years and outcomes at ten years, both for hypothesis (H1) and (H2). In addition, we conducted a secondary analysis for hypothesis (H1), in which mediators (DNAm) were measured at ten years and outcome (aeroallergen sensitization) at 15 years (Fig. 1 and Additional file 1: Figure S1).

Mediation analyses rely on the following three assumptions [28]: (1) no exposure-mediator confounding, (2) no mediator-outcome confounding and (3) no exposure-outcome confounding. To fulfill these assumptions to the best of our knowledge, we constructed directed acyclic graphs (DAGs) to visualize each of these paths using *dagitty* [29] (Additional file 1: Figures S2–S9). A minimal sufficient adjustment set was identified for each pathway via the tracing of association directions and elimination of any potential confounders already associated with a precursory confounder. Exposure-mediator models were adjusted for SES (Exposure: maternal smoking during pregnancy), SES and NO_2_ exposure at birth (family history) and sex (PRS) for both hypotheses. Mediator-Outcome models were adjusted for all potential confounders according to the DAGs (Additional file 1: Figures S2–S9). A detailed description of the definition and assessment of these covariates is provided in Additional file 1: Table S1.

Associations with continuous outcomes (MRS or DNAm in individual CpG sites) were analyzed using linear regression and associations with binary outcomes (allergic sensitization) were analyzed using logistic regression.

### Causal mediation analysis of MRS

Causal mediation analysis, using the R package *mediation* [30], was applied to test the two hypotheses (H1) and (H2) for allergy-related MRS. Results were adjusted for multiple testing using the Benjamini–Hochberg procedure [31] for false-discovery rate (FDR) together within each H1 and H2.

### High-dimensional mediation analysis of individual CpGs

High-dimensional mediation analyses (HMA) were used to test the two hypotheses for individual CpGs. H1 was tested using the Divide-Aggregate Composite-Null test (DACT), HIMA, and gene-based HMA (gHMA). H2 was tested using only DACT, because HIMA and gHMA are only applicable for high-dimensional mediators but not for high-dimensional outcomes.

1. Previous knowledge + Divide-Aggregate Composite-Null test (DACT)
  Based on previously published EWAS of total IgE [1, 2], aeroallergen sensitization [3, 4], childhood asthma [5] and any allergic disease [6] we used existing knowledge on allergy-relevant CpGs to reduce the multiple testing burden. Of the 1673 previously reported CpGs, 1501 were available in the LISA cohort and 583 CpGs were significantly associated with aeroallergen sensitization in the LISA cohort at six years [7] (False discovery rate ≤ 0.05; adjustment for Houseman cell -type estimates to resemble the initial discovery analyses), which were further taken as testing-set of potential mediators. Of note, none of these CpGs were significantly associated with any of the exposures after multiple testing correction and adjustment for sex, detailed age and *EpiDISH* cell-type estimates (Additional file 2: Table S2).
  We used DACT for the composite null hypothesis of no mediation effect as suggested by Liu et al. [32] to improve the multiple testing burden. In short, DACT takes the *p *values from the exposure-mediator and the mediator-outcome model to compute a new joint list of *p*-values, which will be used to determine significance (*p*-value < 0.05). This is done by aggregating the weighted *p* -values of the three possible null-hypotheses leading to no mediation effect and calibrating this using Efron’s empirical null framework [33].
2. HIMA
  Whereas the previous approach relied on existing knowledge as a baseline selection of mediators, HIMA as proposed by Zhang et al. [34] uses a three step procedure to identify significant CpGs throughout the whole epigenome. First, the top CpGs with the largest effect sizes (beta of standardized inputs) for the response variable are identified using sure independence screening (SIS) [35]. The total number of top hits (*N*) varies per model and is calculated by *N* = 2*n/log(*n*), with *n* being the input sample size. To capture relevant CpGs with our smaller sample size, we applied a looser threshold than the original publication. In a second step, HIMA estimates the mediation effect using minimax concave penalty and performs joint significance testing as a third and final step.
3. Gene-based HMA (gHMA)
  We further applied gene-based high-dimensional mediation analysis (gHMA) as proposed by Fang et al. [36]. The idea behind this approach is that not single CpGs but genes act as biological units and should therefore be analyzed together. The functions further provide different modeling options for linear or nonlinear relationships and an omnibus-test to combine both, which outperformed the single models in their simulation study. First, we annotated every CpG to their nearest gene within 20,000 base pairs as done previously [37], resulting in 40,916 different genes. We then applied gHMA to each of these 40,916 genes, each covering between one and 1758 CpGs, performing the linear, nonlinear and omnibus-test for significance. We used differing kernel-thresholds of 0.7, 0.8 and 0.9 as values for explained variance by the kernel principal components. Results of the omnibus-test were corrected using the Benjamini–Hochberg procedure [31].

### Validation of CpG sites using causal mediation analysis

All significant CpG sites identified with the three described methods above are followed up using a causal mediation analysis to determine the direct, indirect, and total effects as well as the proportion mediated. Multiple testing correction followed the one applied for the MRS evaluation by calculating the FDR for all H1 CpGs together, the same correction was applied for H2 CpGs. Models and adjustment are the same as for MRS analyses and single CpGs were afterwards annotated using mQTL databases provided by Gaunt and Hawe et al. [38, 39].

### Sensitivity analyses

We conducted a set of sensitivity analyses to evaluate the robustness of associations for any CpG sites that were successfully validated in the causal mediation analysis described above.

First, to further evaluate the impact of differences in cell-type proportions on our findings, we conducted a sensitivity analysis in which we additionally adjusted all exposure-mediator associations for estimated cell types, which are otherwise only included in the mediator-outcome associations.

Second, to focus exclusively on newly developed aeroallergen sensitization in our mediation analyses with aeroallergen sensitization as outcome, we conducted a sensitivity analysis in which we excluded individuals already sensitized at baseline DNAm measurement.

Third, we conducted sex-stratified analyses, as puberty may play a role in allergen sensitization [40].

### Replication of potential mediators

Single CpGs moving forward to validation in causal mediation analysis was further replicated in the independent Swedish BAMSE (Swedish abbreviation for Children, Allergy, Milieu, Stockholm, Epidemiology) cohort, which recruited 4093 newborns between 1994 and 1996. Ethical approval was given by the Regional Ethics Board (EPN) and further information is available elsewhere [41]. Here, we used exposure data from birth (maternal smoking in second and/or third trimester of pregnancy, any family history of allergic diseases and the same calculated PRS for any allergic disease [7, 23]), DNAm data measured at eight years of age with the Illumina Infinium HumanMethylation450 BeadChip (Illumina Inc., San Diego, USA) [6] and outcome data (positive aeroallergen sensitization to the SX1 mix) from 16 years. Further information on genetic and DNAm data can be found in Additional file 1: Methods S1.

All analyses were performed in R [42] V.4.1.2 in LISA and V.4.1.3 in BAMSE.

## Results

The total sample size for the six different models and time windows, from six to ten years (A) and from ten to 15 years (B), varied from 143 to 229, only including participants, who had all necessary data available (respective exposure, DNAm and covariates) (Fig. 1 and Additional file 1: Figure S1). Participants in the overall sample for all models were majority male (57.7%) and their blood samples were collected primarily during the allergy season from March to August. Prevalence of aeroallergen sensitization increased from baseline to follow-up in each time window and missing values for exposures ranged from six (maternal smoking) to twelve missing values in the PRS (Table 1).

**Table 1:** Description of total sample of LISA participants included in this study

	Time window A (6 and 10 years)	Time window B (10 and 15 years)
Total sample size—*N*	234	227
Confounder		
Male sex—*N* (%) [Nmiss]	135 (57.7%) [0]	131 (57.7%) [0]
Exact age **at baseline**—Mean (sd) [Nmiss]	6.1 (0.2) [0]	10.2 (0.1) [0]
Exact age **at follow-up**—Mean (sd) [Nmiss]	10.2 (0.1) [0]	15.2 (0.2) [52]
Blood taken in allergy season **at baseline**—*N* (%) [Nmiss]	158 (67.5%) [0]	116 (51.1%) [0]
Blood taken in allergy season **at follow-up**—*N* (%) [Nmiss]	124 (53%) [0]	90 (51.4%) [52]
BMI measured **at baseline**—Mean (sd) [Nmiss]	15.3 (1.3) [1]	17.0 (2.5) [1]
BMI measured **at follow-up**—Mean (sd) [Nmiss]	16.9 (2.5) [1]	20.3 (2.8) [59]
High parental education—*N* (%) [Nmiss]	186 (80.2%) [2]	181 (80.4%) [2]
N0_2_ at birth address—Mean (sd) [Nmiss]	21.4 (6.1) [1]	21.2 (5.5) [1]
Exposures		
(i) Maternal smoking during pregnancy—*N* (%) [Nmiss]	17 (7.5%) [7]	16 (7.2%) [6]
(ii) At least one parent allergic—*N* (%) [Nmiss]	151 (64.5%) [0]	148 (65.2%) [0]
(iii) PRS—Mean (sd) [Nmiss]	0.2 (1.0) [12]	0.2 (0.9) [11]
Outcome		
Sensitized **at baseline**—*N* (%) [Nmiss]	74 (31.6%) [0]	101 (44.5%) [0]
Sensitized **at follow-up**—*N* (%) [Nmiss]	105 (44.9%) [0]	84 (50.3%) [60]

Baseline is defined as the first time point of the model (six or ten) and follow-up as the second (ten or 15, respectively). The sample sizes for the mediation models with different exposures were as followed: Hypothesis 1A—*N*_MaternalSmoking_ = 215, *N*_FamilyHistory_ = 198, *N*_PRS_ = 211; Hypothesis 1B—*N*_MaternalSmoking_ = 163, *N*_FamilyHistory_ = 154, *N*_PRS_ = 158; Reversed models for Hypothesis 2A (Exposure–Sensitization–DNAm)—*N*_MaternalSmoking_ = 216, *N*_FamilyHistory_ = 222, *N*_PRS_ = 212. See also Additional file 1: Figure S1

### Causal mediation analysis for MRS

**Fig. 2:**
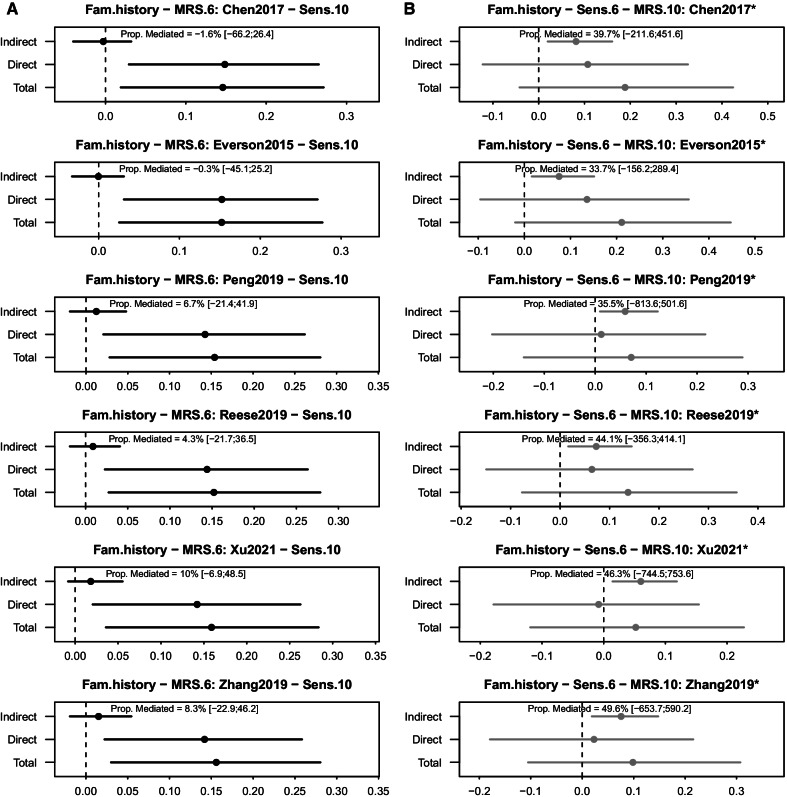
MRS as predictor or consequence of allergic disease. Significant indirect effects are indicated with an asterisk. The title follows the pattern exposure–mediator–outcome. Evaluation **A** whether the association between family history of allergic disease and allergic sensitization at ten years is mediated by prior changes in DNAm at six years (measured by MRS) or **B** whether the association between family history of allergic disease and changes in DNAm at ten years (measured by MRS) is mediated by prior allergic sensitization at six years. The six MRS can be allocated to the following phenotypes: Chen2017—total IgE, Everson2015—atopy, Peng2019—aeroallergen sensitization, Reese2019—childhood asthma, Xu2021—any allergy and Zhang2019—atopy, respectively

Allergy-related MRS were not found to be a mediator of the association between family history of allergies and subsequent allergic sensitization (H1, Fig. 2A). However, we found significant indirect effects for the association between family history of allergies and all six allergy-related MRS with prior allergic sensitization as mediators (H2) (e.g., Indirect effect (Chen2017) = 0.081 [0.020; 0.160]). Proportion mediated by allergic sensitization ranged from 33.7% (Everson2015) to 49.6% (Zhang2019) (Table 2 and Fig. 2B). Results were robust to additional adjustment for cell-type estimates as exposure-mediator confounders in our sensitivity analysis (Additional file 2: Table S3 and S4), while keeping the mediator-outcome confounders, including cell-type estimates, consistent.

**Table 2:** Significant mediation (FDR < 0.05) between family history as exposure and MRS, mediated by aeroallergen sensitization measured (H2)

Outcome	Indirect effect [95% CI]	Direct effect [95% CI]	Total effect [95% CI]	Prop.Med [95% CI]
**(A) Sensitization at 6 and DNAm measured at 10 years**				
*(ii) Family history of allergic disease (both parents allergic)*				
Chen2017 *(IgE)*	0.081 [0.020; 0.160]	0.107 [− 0.122; 0.326]	0.188 [− 0.042; 0.425]	0.397 [− 2.116; 4.516]
Everson2015 *(Atopy)*	0.075 [0.016; 0.151]	0.136 [− 0.096; 0.356]	0.211 [− 0.020; 0.447]	0.337 [− 1.562; 2.894]
Peng2019 *(Aeroallergen)*	0.059 [0.009; 0.123]	0.012 [− 0.202; 0.216]	0.071 [− 0.140; 0.289]	0.355 [− 8.136; 5.016]
Reese2019 *(Asthma)*	0.073 [0.017; 0.145]	0.064 [− 0.149; 0.268]	0.137 [− 0.077; 0.357]	0.441 [− 3.563; 4.141]
Xu2021 *(Allergy)*	0.060 [0.014; 0.119]	− 0.008 [− 0.179; 0.154]	0.052 [− 0.119; 0.227]	0.463 [− 7.445; 7.536]
Zhang2019 *(Atopy)*	0.076 [0.019; 0.148]	0.023 [− 0.179; 0.216]	0.099 [− 0.105; 0.307]	0.496 [− 6.537; 5.902]

No significant associations were found for (i) maternal smoking or (iii) PRS for any allergies

We did not find any significant mediation effects for maternal smoking during pregnancy or the PRS for either of the two hypotheses. Full results for all MRS models can be found in Additional file 2: Tables S5 (H1) and S6 (H2) for the time window from six to ten only, as DNAm as an outcome was not measured at 15 years of age.

### DACT

**Fig. 3:**
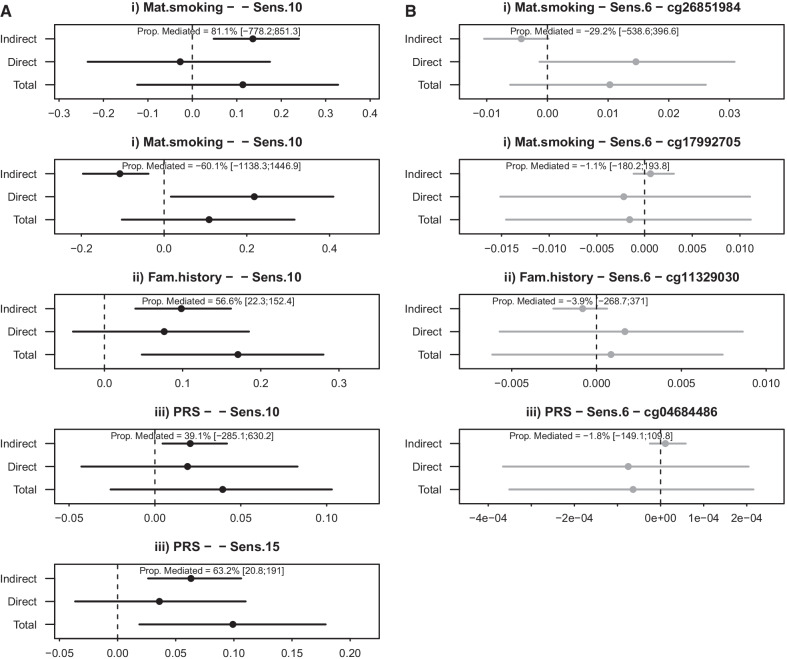
DNAm in individual CpG sites as predictor or consequence of allergic disease. CpG sites that were identified as mediators in at least one HMA (HIMA or DACT) and validated in causal mediation analysis are presented. Significant indirect effects are indicated with an asterisk and the title follows the pattern exposure–mediator–outcome. Evaluation **A** whether the association between (i) maternal smoking during pregnancy/(ii) family history of allergic disease/(iii) PRS for any allergies and allergic sensitization at ten/15 years is mediated by prior changes in DNAm at six/ten years or **B** whether the association between (i) maternal smoking during pregnancy/(ii) family history of allergic disease/(iii) PRS for any allergies and changes in DNAm at ten years is mediated by prior allergic sensitization at six years. For cg19310430 there is no corresponding model for hypothesis (H2) as DNAm was not measured at 15 years

We identified 90 unique CpGs as potential mediators (H1) with the DACT approach: For the first time window (A) from six to ten years, we found 18 CpGs for maternal smoking, 51 for family history and six for the PRS. For the second time window from ten to 15 the numbers were 20, 19 and ten, respectively. Of all of these, only one CpG (cg26851984) was validated in causal mediation analyses (significant indirect effect after multiple-testing correction), for time window A and maternal smoking as exposure (Table 3). Differential DNAm at cg26851984 mediates 81% of the association between maternal smoking and aeroallergen sensitization and is robust to additional adjustment for cell-type estimates of the exposure-mediator association. Of note, cg26851984 is also an mQTL with 58 surrounding SNPs as reported in a recent publication by Hawe et al. [39]. A mediation plot for cg26851984 is presented in Fig. 3 (first panel) showing the validated associations with the single CpG as mediators.

**Table 3:** DNAm in individual CpG sites as predictors of aeroallergen sensitization (H1). Displayed CpGs were significantly validated in the causal mediation analysis (FDR < 0.05)

HMA Method	Mediator [CpG (UCSC/nearest gene—UCSC Group)]	Indirect effect [95% CI]	Direct effect [95% CI]	Total effect [95% CI]	Prop.Med [95% CI]	mQTL (Hawe et al.^a^)
**A. DNAm at six years and sensitization measured at ten years**						
*(i) Maternal smoking during pregnancy*						
DACT	cg26851984 *(/RP11-772E11.1)*	0.139 [0.050; 0.242]	− 0.025 [− 0.226; 0.182]	0.114 [− 0.124; 0.328]	0.811 [− 7.782; 8.513]	58
HIMA	cg17992705 *(ATXN2L - ExonBnd)*	− 0.108 [− 0.193; − 0.041]	0.217 [0.017; 0.397]	0.109 [− 0.102; 0.315]	− 0.601 [− 11.384; 14.469]	
*(ii) Family history of allergic disease (both parents allergic)*						
HIMA	cg11329030 *(/ATP6V1E1P1)*	0.095 [0.040; 0.158]	0.079 [− 0.025; 0.193]	0.174 [0.060; 0.280]	0.547 [0.218; 1.349]	
*(iii) PRS for any allergies*						
HIMA	cg04684486 *(SLC31A2 - TSS200)*	0.020 [0.004; 0.042]	0.019 [− 0.043; 0.082]	0.040 [− 0.026; 0.103]	0.390 [− 2.851; 6.302]	
**B. DNAm at ten years and sensitization measured at 15 years**						
(iii) PRS for any allergies						
HIMA	cg19310430 *(C11orf45 - 5'UTR)*	0.063 [0.026; 0.105]	0.036 [− 0.036; 0.110]	0.099 [0.019; 0.179]	0.632 [0.208; 1.910]	

CpG sites that were identified as mediators in at least one high-dimension mediation analysis (HMA) method (HIMA or DACT) and validated in causal mediation analysis (significant indirect effect) are presented.

^a^No mQTLs from Gaunt et al. [38] were matched to the respective CpGs

In the reversed models investigating sensitization as a potential mediator of subsequent changes in DNAm (H2), we did not identify any mediation effects for individual CpGs in either main model (Additional file 2: Table S7).

### HIMA

Dependent on the sample size of the different exposures and time windows, between 58 and 85 CpGs (*N* = 2*n/log(*n*); Fig. 1) were screened for highest effect sizes during the first step of HIMA and had their estimates calculated and tested for joint significance in HIMA in different models. We identified three CpGs as potential mediators in the time window from six to ten years (time window A), one CpG of the association between each exposure and aeroallergen sensitization. In addition, we identified four CpGs as mediators in the later time window (B) from ten to 15 years, three for PRS as exposure and one for family history (Additional file 2: Tables S7 for full results and S8 for annotated hits). Four of the seven identified CpGs were significantly validated in the causal mediation analysis and none are located in mQTLs (Table 3; Fig. 3 (panels 2–5)).

### Sensitivity analyses

All CpGs presented in Table 3 showed nominal significant associations after additional adjustment for cell-type proportions between exposure and mediator (Additional file 2: Table S9) and when restricting the analysis sample to those who were not sensitized at the time of DNAm measurement (Additional file 2: Table S10). However, those associations were not significant after adjustment for multiple testing. We did not find sex-specific differences in mediation effects in terms of effect estimates and direction of effects, but indirect effects were only significant for three of the five CpGs in males (Additional file 2: Table S11) and for none of the CpGs in females (Additional file 2: Table S12), most likely due to the reduced sample size.

### gHMA

We did not identify any significant genes for either time window or exposure with the gHMA method.

### Replication in BAMSE

Data was available for 445 participants with DNAm measured at eight and aeroallergen sensitization measured at 16 years of age (Additional file 2: Table S13). Table 4 presents the results from BAMSE for our previously validated CpGs (Table 3). Due to the different arrays used in LISA and BAMSE, only two of the five CpGs were available for replication. None of these two CpGs could be replicated in BAMSE, but for cg26851984 the directions of the indirect and direct effects are the same compared to LISA. Full results are included in Additional file 2: Table S14.

**Table 4:** DNAm in individual CpG sites as predictors of aeroallergen sensitization (H1). Replication of validated CpGs (Table 3) in BAMSE

HMA Method	Mediator [CpG (UCSC/nearest gene - UCSC Group)]	Indirect effect [95% CI]	Direct effect [95% CI]	Total effect [95% CI]	Prop.Med [95% CI]	mQTL (Hawe^a^)
**A. DNAm at eight and sensitization measured at 16 years**						
*(i) Maternal smoking during pregnancy*						
DACT	cg26851984 *(/RP11-772E11.1)*	0.004 [− 0.012; 0.025]	− 0.035 [− 0.193; 0.134]	− 0.031 [− 0.189; 0.138]	− 0.001 [− 1.667; 1.304]	58
*(iii) PRS for any allergies*						
HIMA	cg19310430 *(C11orf45 - 5'UTR)*	0.000 [− 0.004; 0.004]	0.101 [0.048; 0.154]	0.101 [0.050; 0.154]	0.000 [− 0.041; 0.050]	

CpGs cg17992705, cg11329030 and cg04684486 are only available on the EPIC array and thus not available in BAMSE, which used the 450K array.

^a^No mQTLs from Gaunt et al. were matched to the respective CpGs

## Discussion

The present study investigated whether DNAm is a potential cause/predictor or a consequence/outcome of sensitization by conducting causal mediation analyses for well-known risk factors of aeroallergen sensitization as exposures (maternal smoking during pregnancy, family history of allergies, and PRS for any allergy) and data on DNAm and aeroallergen sensitization from two consecutive time points as outcomes. We found evidence that DNAm in most previously identified CpG sites (summarized in MRS) was a consequence rather than a cause of aeroallergen sensitization. In addition, we identified five single CpGs that mediated the association between maternal smoking during pregnancy, family history of allergic diseases and a PRS and subsequent aeroallergen sensitization, thus serving as predictors of sensitization. Aggregating both hypotheses, we suggest that DNAm can be a cause as well as a consequence of aeroallergen sensitization, depending on the genomic location.

This study further attempted replication of identified CpGs in the independent Swedish BAMSE cohort but could not significantly replicate any of the five reported CpGs. This might, however, not necessarily negate our findings, as three of the five CpGs were not measured in BAMSE (450K chip vs. EPIC chip in LISA). Furthermore, the time difference is larger between the two assessment points in BAMSE (eight to 16 vs. ten to 15 in LISA). To the best of our knowledge, there are no previous studies investigating causal epigenetic mediation between prenatal exposures and aeroallergen sensitization in childhood and adolescence. Previous studies have reported mediation effects of DNAm for the associations between body-mass-index (BMI) and trajectories with asthma [43], BMI and cardio-metabolic risk [44], and age at puberty onset and lung function [45]. Of note, none of these studies investigated both directions, DNAm as both a predictor (H1) and as a consequence (H2).

Publications investigating mQTLs found that DNAm changes are often seen as a consequence of diseases rather than their cause [46] and this is supported by our findings on the allergy-related MRS. However, in the present study we also identified CpGs which serve as mediators for the association between known determinants of allergies and aeroallergen sensitization. Of note, none of the identified single CpGs are part of the evaluated MRS after clumping and thresholding, even though one has been previously reported by the same EWAS as an associated CpG site (Peng [3]). This might indicate that DNAm acts in both effect directions, represented by differing sets of CpG loci.

On the one hand, our finding that MRS are rather a consequence than a cause of sensitization falls in line with our previous results [7], which might also rely on the fact that the pre-identified CpGs were reported in mostly cross-sectional EWAS. On the other hand, the single CpGs mediating prenatal exposures on aeroallergen sensitization later in life, might be facilitated as early predictors for disease development. These should be followed up in future studies to further determine their clinical relevance.

For cg26851984, which was identified as a mediator of the association between maternal smoking during pregnancy and sensitization with DACT, we identified the closest gene to be *PRPF3*. This gene is associated with eczema [13], eosinophil counts [47] and any allergy [15], supporting the importance of this CpG as a mediator of allergen sensitization. Of note, this CpG was previously reported in an EWAS on aeroallergen sensitization [3], as only previously known CpGs were tested as potential mediators with the DACT method. However, it is not part of the allergy-related MRS previously calculated based on these EWAS [7] after clumping and thresholding. Further, it is a mQTL and its associations have to be interpreted with caution as effects here could be attributable to surrounding SNPs, which may explain the higher mediation effect size (0.139 for maternal smoking as exposure and cg26851984 as mediator) compared to all others (≤ 0.108), but also the higher albeit non-significant proportion mediated of 81.1%.

Other CpGs identified with the hypothesis-generating HIMA approach were also located in proximity to allergy-relevant genes. *ATXN2L*, located in the exon boundary and corresponding to cg17992705, is associated with forced vital capacity [48], a lung function parameter that is reduced in asthma patients. Further, *DIP2C* (cg12724894) and *ASB2* (cg03389164) are associated with eosinophil counts [49, 50] and located within the gene body and promoter, respectively.

Looking at Figs. 2 and 3, it can be seen that not all total effects are significant while the indirect effects are. While significant total effects were a prerequisite of potential mediation in the traditional causal step approach proposed by Baron and Kenny in 1986 [51], it is not a formal requirement in the causal mediation analysis approach we used, but reduces that statistical power to detect indirect effects [50, 51]. While all of our exposures are known risk factors for aeroallergen sensitization, they might not necessarily show significance in our reduced sub-sample. The total effect is defined as the sum of the direct and all indirect effects and we do sometimes observe opposite effect signs for direct and indirect effects (e.g., cg17992705), which can attenuate the total effects.

The present study has multiple strengths: We have objectively measured data on all levels of the analysis for the model in which PRS is the exposure, as neither PRS, DNAm, nor blood-measured aeroallergen sensitization is subject to recall bias. In addition, the LISA study is a well-established prospective German birth cohort with still ongoing follow-up and provides a valuable data source for studying allergic diseases. This also supports the causal interpretation, as the longitudinal succession of measured mediators and outcomes was possible due to the longitudinal design of the study. DNAm is being measured repeatedly at both six and ten years, as well as consecutive time points being used for the definition of exposure, mediator, and outcome. This longitudinal design might also enable future analyses, ideally paired with similar studies with comparable design to reach higher statistical power for epigenome-wide mediation analyses. Further, we applied three different HMA methods complemented with causal mediation analysis to investigate their applicability to the allergic context in contrast to simpler screening methods for reduction of the multiple-testing burden. Each HMA approach is based on different assumptions and uses different strategies to deal with the challenges of multiple testing.

Limitations of the presented study include the small sample size, which might be insufficient to detect all potential mediation effects, especially as effects of single CpGs are rather small. This might also explain why we could not replicate single CpGs in both time windows (A&B) or why we did not find significant gene-units using the gHMA approach. It could also be speculated that single CpGs might be more relevant in relation to allergic sensitization than methylation across a whole gene, as this is the biggest difference between gHMA as a gene-based approach and the others (HIMA and DACT) as CpG-based approaches. Further, applying the PRS as an exposure, we did not check whether there is significant mediation between single SNPs and CpGs, but with the development of relevant methodology [52] this is of great interest for future studies. MRS were further determined according to their cross-sectional prediction accuracy and not optimized according to their performance in a prospective or mediation setting as applied here. Another general issue might be confounding, which is a serious problem in mediation analysis [28]. We adjusted our models based on DAGs to the best of our knowledge, however, unmeasured confounding cannot be ruled out completely in observational studies.

## Conclusions

In conclusion, we found indications that DNAm could either be the cause of allergic sensitization or the consequence thereof, depending on the genomic location. The two different sets of DNAm patterns, namely MRS as consequence of sensitization or single CpGs as cause, have differing clinical implications: While MRS might be considered as cross-sectional biomarkers, the single CpGs might be clinically relevant early predictors of sensitization and should be investigated in future studies.

## Supplementary Information

**Additional file 1**. Supplementary methods, Table S1 and Figures S1-S9.

**Additional file 2**. Supplementary Tables S2-S14.

## Data Availability

Due to data protection reasons, the datasets generated and/or analyzed during the current study cannot be made publicly available. The datasets are available to interested researchers from the corresponding author on reasonable request, provided the release is consistent with the consent given by the LISA and/or BAMSE study participants. Ethical approval might be obtained for the release and a data transfer agreement from the legal department of the Helmholtz Zentrum München and/or Karolinska Institutet must be accepted.

## Abbreviations

BAMSE: Barn (= Children) Allergy Milieu Stockholm Epidemiology
BMI: Body Mass Index
CpG: Cytosine and Guanine only separated by their phosphate backbone
CTP: Cell-type proportions
DACT: Divide-Aggregate Composite-Null Test
DAG: Directed Acyclic Graph
DNAm: DNA methylation
EWAS: Epigenome-wide association studies
FDR: False Discovery Rate
gHMA: Gene-based High-dimensional Mediation Analysis
GWAS: Genome-wide association studies
HMA: High-dimensional mediation analysis
IgE: Immunoglobulin E
LISA: Influence of Life-style factors on Development of the Immune System and Allergies in East and West Germany study
MRS: Methylation Risk Score
NO_2_: Nitrogen Dioxide
PRS: Polygenic risk score
SES: Socio-economic status
SIS: Sure Independence Screening
