# Supplementary material for: DNA methylation and aeroallergen sensitization: The chicken or the egg?
Source: Clin Epigenetics. 2022 Sep 16;14:114. doi: 10.1186/s13148-022-01332-5 (PMC9482323; doi:10.1186/s13148-022-01332-5)
Supplement: Supplementary file 1 — Additional file 1. Supplementary methods, Table S1 and Figures S1-S9. [file 13148_2022_1332_MOESM1_ESM.docx]

# Online supporting information

### List of supplementary information provided

Methods S1: Genetic and DNAm data in BAMSE

Table S1: Definition of Exposures and Confounders (word supplement)

Table S2: Full results for MRS models hypothesis 1

Table S3: Full results for MRS models hypothesis 2

Table S4: Full results for MRS models hypothesis 1 – sensitivity analysis

Table S5: Full results for MRS models hypothesis 2 – sensitivity analysis

Table S6: Full results for CpG models hypothesis 1

Table S7: Full results for CpG models hypothesis 2

Table S8: Full results for CpG models hypothesis 1 – sensitivity analysis

Table S9: Full results for CpG models hypothesis 1 – sensitivity analysis

Table S10: HIMA CpGs and corresponding genes

Plot S1: Flow-chart of models

Plots S2-S9: Directed acyclic graphs for confounder selection

Methods S1: Genetic and DNAm data in BAMSE

Results for this study were replicated in the independent BAMSE cohort. These supplementary methods provide more detailed information about applied methods for genotyping (Exposure PRS) and DNAm measurement (Mediators), as well as the outcome aeroallergen sensitization.

Within the BAMSE project, genotyping was done on the Illumina Human 610-quad array (Illumina, Inc., San Diego, CA). A total of 505 samples were genotyped (a subset of the study consisting of asthma cases and controls, out of which 485 samples were of good genotype quality. Samples were further excluded if the 10 genetic principal components indicated a non-EU ethnic outlier after projection of the study samples on the 1000 Genomes reference sample. For imputation, samples were also excluded if their genotyping success rate was lower than 95% and SNPs were excluded for - Call rate < 95%; HWE p< 1e-6; MAF < 0.01, determined using Plink 2.0. After further quality control a total of 464 participants within wave1 and 2194 participants within wave2 remained. All data was imputed using the HRC version 1.1 (1) on the Sanger imputation server (EAGLE2+PBWT) for BAMSE. Further information on the PRS calculation are described elsewhere (2).

For this methylation study, we used data from the 8-year follow-up. At 8 years, epigenome-wide DNA methylation was measured in 472. 500 ng DNA per sample underwent bisulfite conversion using the EZ-96 DNA Methylation kit (Zymo Research Corporation, Irvine, USA). Samples were plated onto 96-well plates in randomized order. Samples were processed with the Illumina Infinium HumanMethylation450 BeadChip (Illumina Inc., San Diego, USA). Quality control of analyzed samples was performed using standardized criteria. Samples were excluded in case of sample call rate <99%, colour balance >3, low staining efficiency, poor extension efficiency, poor hybridization performance, low stripping efficiency after extension and poor bisulfite conversion. We also applied multidimensional scaling (MDS) plot to evaluate gender outliers based on chromosome X data, that produced two separated clusters for male and female. Samples that did not belong to the distinct cluster were removed. Furthermore, we applied median intensity plot for methylated and unmethylated intensity by using the minfi R package (samples below the 10.5 cutoff were excluded). Applying these criteria resulted in exclusion of 8 samples. Probes with a single nucleotide polymorphism in the single base extension site with a frequency of >5% were excluded [4], as were probes with non-optimal binding (non-mapping or mapping multiple times to either the normal or the bisulphite-converted genome), and the probed belonging to chr X and chr Y, resulting in the exclusion of 46,799 probes, leaving a total of 438,713 probes in the analyses. Furthermore, we implemented “DASEN” recommended from wateRmelon package to do signal correction and normalization (3) and covariate batch was also accounted in the model, based on the bisulfite treatment date. Detailed information could be found elsewhere (4).

IgE measures for the outcome were obtained at eight and 16 years using the ImmunoCAP System (Thermo Fisher Scientific, Uppsala, Sweden). IgE-sensitization to allergens was defined as IgE ≥ 0.35 kU/l to one or more of the common tested airborne allergens.

Table S1: Definition of Exposures and Confounders

| **Exposure** | **Definition** | **Timepoints** | **Report in literature (for confounders)** |
| --- | --- | --- | --- |
| Maternal smoking | Maternal smoking during the second and/or third trimester of pregnancy; control defined as stopped smoking before the second trimester or never smoked | Determined pre-natally; assessed at birth questionnaire |  |
| Family history | Dummy whether no or at least one biological parent has ever had any allergic disease (asthma, dermatitis, allergic rhinitis) | Determined pre-natally; assessed at birth questionnaire |  |
| PRS | Polygenic risk score for any allergic disease as calculated from Ferreira et al. 2017&2019(5,6) | Determined at conception; blood or saliva samples collected at six or ten years |  |
| **Confounder** |  |  |  |
| Sex | Biological sex of the participants |  | King 2022(7) |
| Age | Exact age at blood withdrawal | At blood-withdrawal/physical examination | Shahal 2022(8), Horvarth 2013(9) |
| Allergy season | Dummy, whether blood withdrawal took place within the allergy season (March to August) | At blood-withdrawal/physical examination | North 2018(10) |
| CTPs | Cell type proportions as estimated with the *EpiDish* package(11); include CD8+ T-cells, CD4+ T-cells, Natural Killer (NK) cells, B-cells, Monocytes. Neutrophils, Eosinophils | At blood-withdrawal/physical examination | Houseman 2012(12), Seumois 2020(13) |
| BMI | Body mass index as calculated from height and weight measured at physical investigation | At blood-withdrawal/physical examination | Rzehak 2019(14), Wahl 2017(15) |
| SES | Socio economic status depicted here by parental education low/middle (= less or equal than 9 years of education) versus high education (=more than 9 years) | Assessed at birth questionnaire | Cerutti 2021(16) |
| Air pollution | NO_2_ as proxy for general air pollution estimated at birth address within the ESCAPE project (described elsewhere(17)) | Assessed at birth | Park 2022(18), Aguilera 2022(19) |

Plots S1: Flow-chart of models


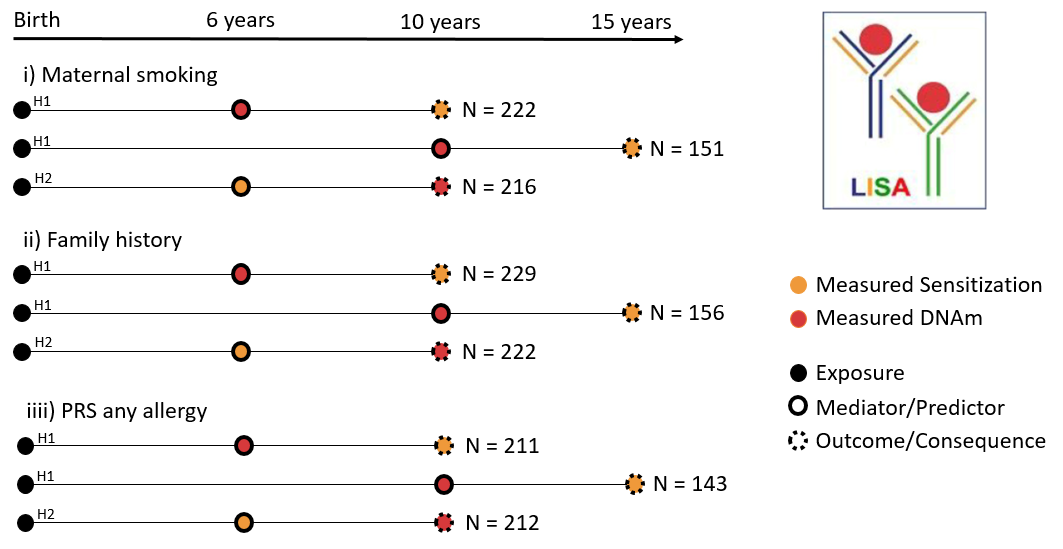


Figure S1: Model display per exposure (i-iii) and hypotheses. **H1)** Hypothesis 1 investigating whether prior DNAm influences sensitization, **H2)** Hypothesis 2 investigating whether sensitization influences posterior DNAm changes.

Plots S2-S9: Directed acyclic graphs for selection of confounders

In the present study confounders for mediation analyses were selected based on directed acyclic graphs (DAGs) created with *dagitty*(20)*.*

The package annotates the DAGs as followed. Paths: Causal paths (green), biasing paths (pink). Nodes: Ancestors of exposure (yellow), ancestors of the outcome (blue), ancestors of both exposure and outcome (pink).

They are ordered according to the two tested hypotheses (H1: Exposure – DNAm – Sensitization & H2: Exposure – Sensitization – DNAm) and the three different exposures (i) Maternal smoking, ii) Family history and iii) PRS), while the first plot for each hypothesis always depicts the mediator-outcome association, which is the same for all exposure models in this effect direction.

**Hypothesis (1) (Exposure – DNAm – Sensitization)**

*Mediator-Outcome*


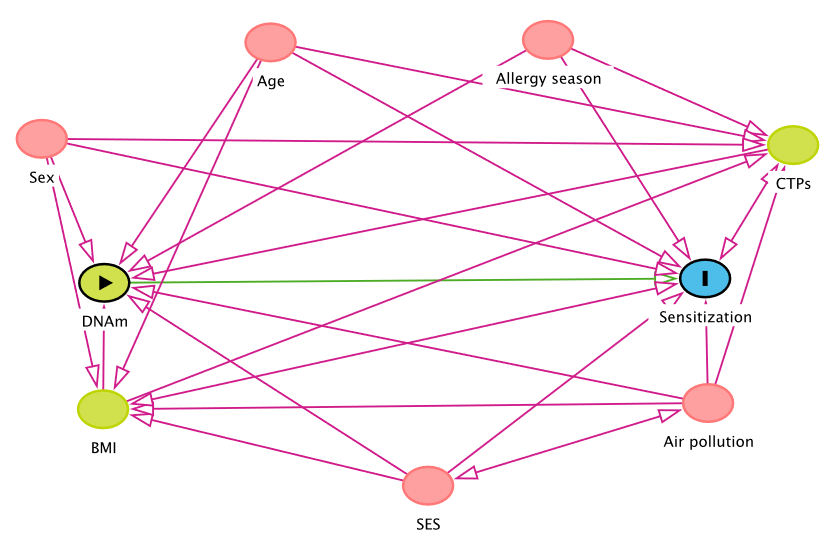


Adjust for: Age, Air pollution, Allergy season, BMI, Cell type proportions (CTPs), SES, Sex

*Exposure-Mediator*

1. Maternal smoking


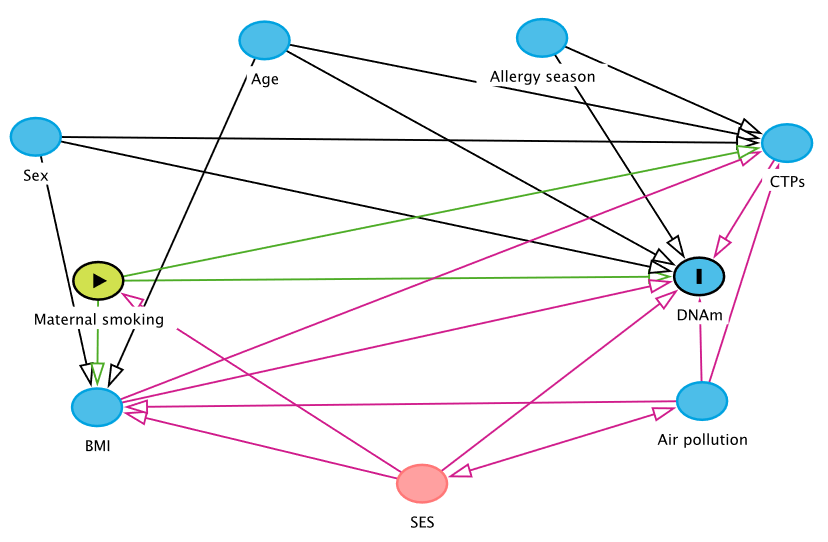


Adjust for: SES

1. Family history


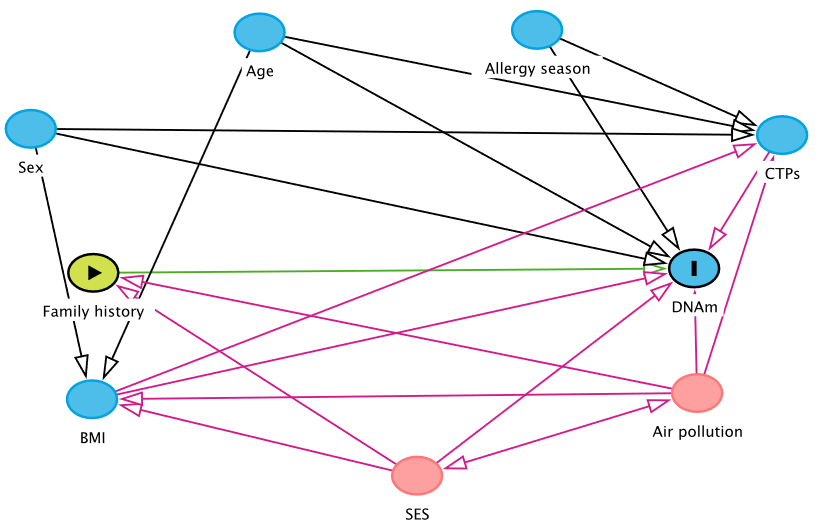


Adjust for: SES, Air pollution

1. PRS


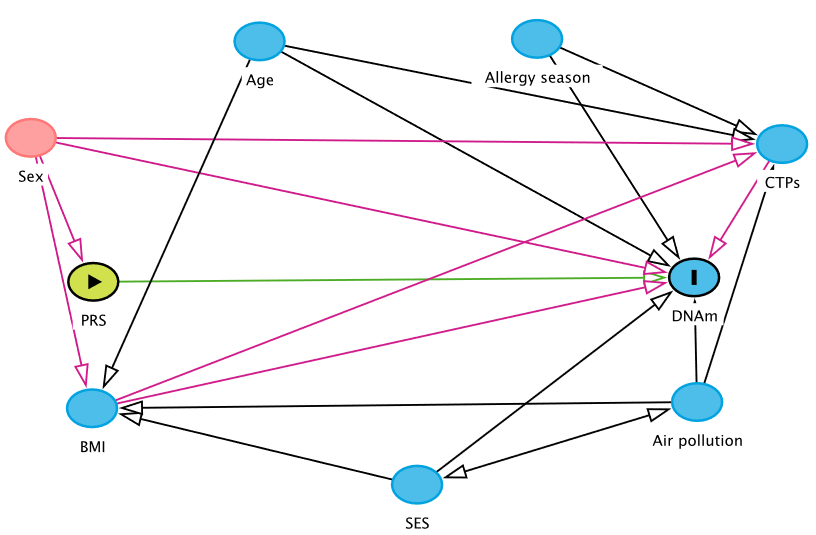


Adjust for: Sex

**Hypothesis (2) (Exposure –Sensitization - DNAm)**

*Mediator-Outcome*


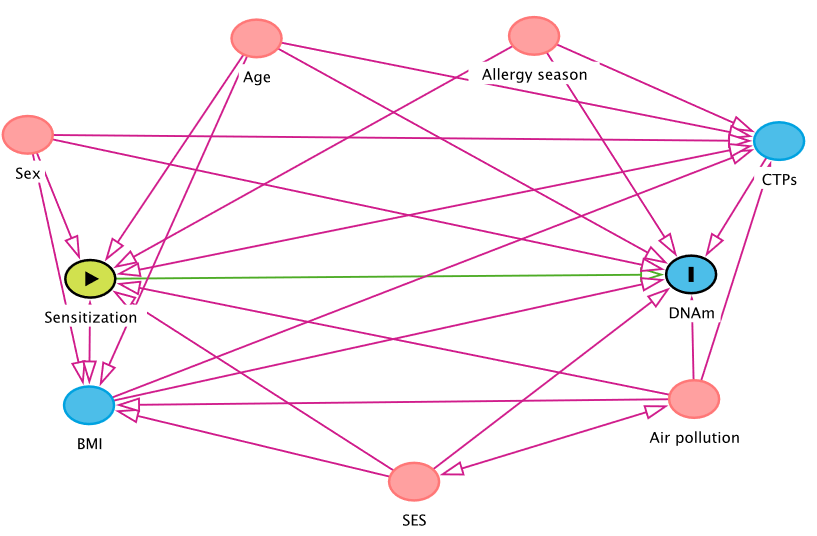


Adjust for: Age, Air pollution (NO_2_), Allergy season, BMI, Cell type proportions (CTPs), SES, Sex

*Exposure-Mediator*

1. Maternal smoking


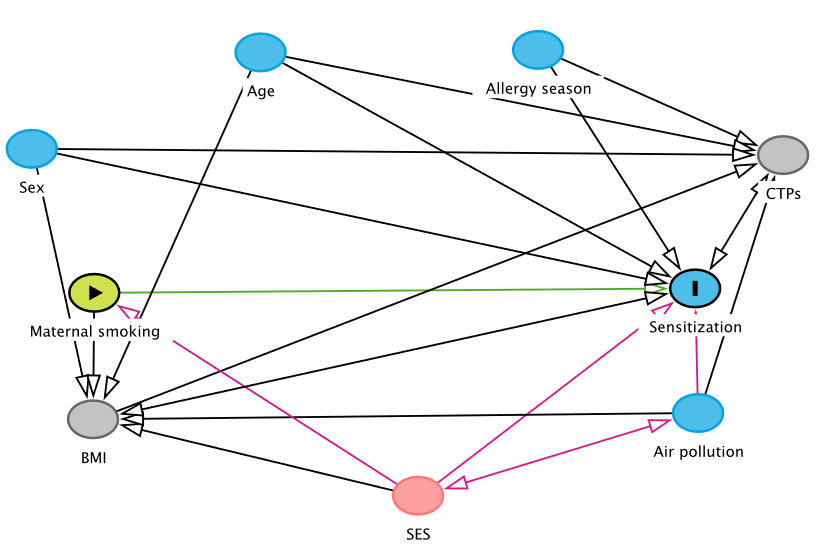


Adjust for: SES

1. Family history


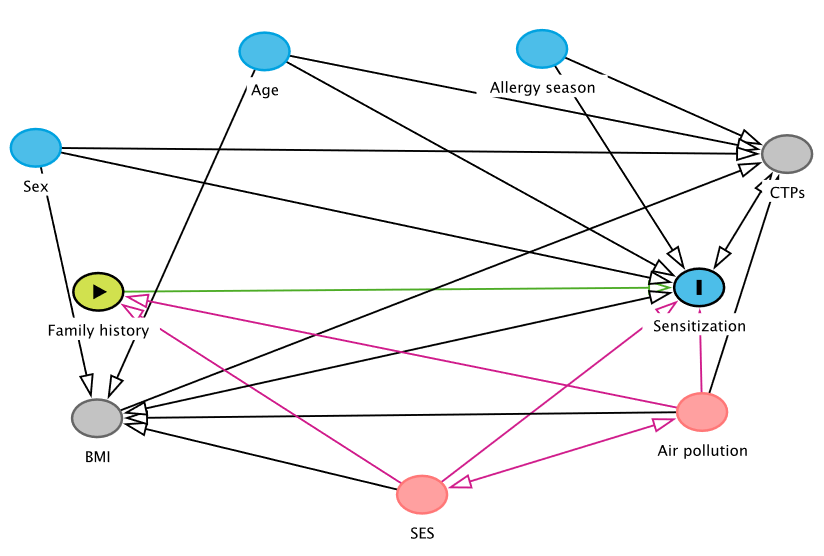


Adjust for: SES, Air pollution

1. PRS


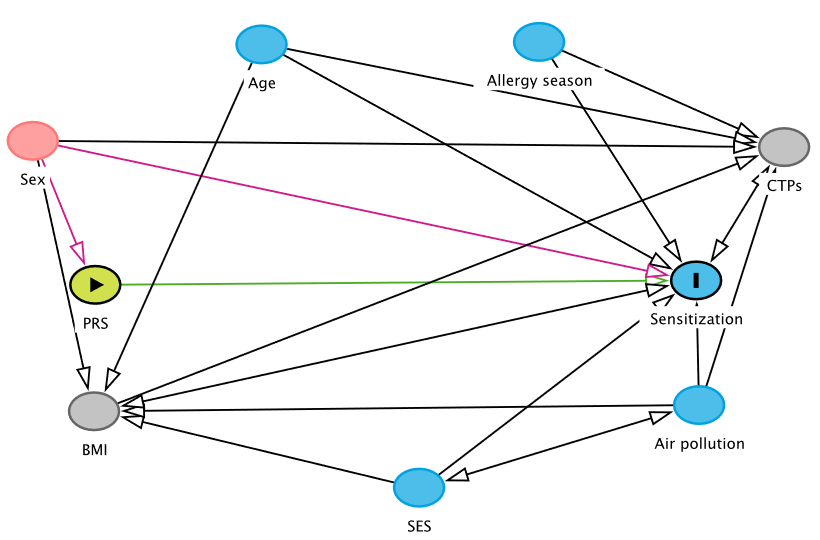


Adjust for: Sex

References

1. McCarthy S, Das S, Kretzschmar W, Delaneau O, Wood AR, Teumer A, et al. A reference panel of 64,976 haplotypes for genotype imputation. Nat Genet. 2016 Oct;48(10):1279–83.

2. Kilanowski A, Chen J, Everson T, Thiering E, Wilson R, Gladish N, et al. Methylation risk scores for childhood aeroallergen sensitization: Results from the LISA birth cohort. Allergy [Internet]. [cited 2022 Apr 27];n/a(n/a). Available from: https://onlinelibrary.wiley.com/doi/abs/10.1111/all.15315

3. Pidsley R, Y Wong CC, Volta M, Lunnon K, Mill J, Schalkwyk LC. A data-driven approach to preprocessing Illumina 450K methylation array data. BMC Genomics. 2013 May 1;14:293.

4. Merid SK, Novoloaca A, Sharp GC, Küpers LK, Kho AT, Roy R, et al. Epigenome-wide meta-analysis of blood DNA methylation in newborns and children identifies numerous loci related to gestational age. Genome Med. 2020 Mar 2;12(1):25.

5. Ferreira MA, Vonk JM, Baurecht H, Marenholz I, Tian C, Hoffman JD, et al. Shared genetic origin of asthma, hay fever and eczema elucidates allergic disease biology. Nat Genet. 2017 Dec;49(12):1752–7.

6. Ferreira MAR, Vonk JM, Baurecht H, Marenholz I, Tian C, Hoffman JD, et al. Eleven loci with new reproducible genetic associations with allergic disease risk. J Allergy Clin Immunol. 2019 Feb 1;143(2):691–9.

7. King DE, Sparling AC, Lloyd D, Satusky MJ, Martinez M, Grenier C, et al. Sex-specific DNA methylation and associations with in utero tobacco smoke exposure at nuclear-encoded mitochondrial genes. Epigenetics. 2022 Mar 3;1–17.

8. Shahal T, Segev E, Konstantinovsky T, Marcus Y, Shefer G, Pasmanik-Chor M, et al. Deconvolution of the epigenetic age discloses distinct inter-personal variability in epigenetic aging patterns. Epigenetics Chromatin. 2022 Mar 7;15(1):9.

9. Horvath S. DNA methylation age of human tissues and cell types. Genome Biol. 2013 Dec 10;14(10):3156.

10. North ML, Jones MJ, MacIsaac JL, Morin AM, Steacy LM, Gregor A, et al. Blood and nasal epigenetics correlate with allergic rhinitis symptom development in the environmental exposure unit. Allergy. 2018 Jan;73(1):196–205.

11. Teschendorff AE, Breeze CE, Zheng SC, Beck S. A comparison of reference-based algorithms for correcting cell-type heterogeneity in Epigenome-Wide Association Studies. BMC Bioinformatics. 2017 Feb 13;18:105.

12. Houseman EA, Accomando WP, Koestler DC, Christensen BC, Marsit CJ, Nelson HH, et al. DNA methylation arrays as surrogate measures of cell mixture distribution. BMC Bioinformatics. 2012 May 8;13:86.

13. Seumois G, Ramírez-Suástegui C, Schmiedel BJ, Liang S, Peters B, Sette A, et al. Single-cell transcriptomic analysis of allergen-specific T cells in allergy and asthma. Sci Immunol. 2020 Jun 12;5(48):eaba6087.

14. Rzehak P, Covic M, Saffery R, Reischl E, Wahl S, Grote V, et al. DNA-Methylation and Body Composition in Preschool Children: Epigenome-Wide-Analysis in the European Childhood Obesity Project (CHOP)-Study. Sci Rep [Internet]. 2017 Oct 30 [cited 2019 Oct 9];7. Available from: https://www.ncbi.nlm.nih.gov/pmc/articles/PMC5662763/

15. Wahl S, Drong A, Lehne B, Loh M, Scott WR, Kunze S, et al. Epigenome-wide association study of body mass index, and the adverse outcomes of adiposity. Nature. 2017;541(7635):81–6.

16. Cerutti J, Lussier AA, Zhu Y, Liu J, Dunn EC. Associations between indicators of socioeconomic position and DNA methylation: a scoping review. Clin Epigenetics. 2021 Dec 14;13(1):221.

17. MacIntyre EA, Gehring U, Mölter A, Fuertes E, Klümper C, Krämer U, et al. Air pollution and respiratory infections during early childhood: an analysis of 10 European birth cohorts within the ESCAPE Project. Environ Health Perspect. 2014 Jan;122(1):107–13.

18. Park J, Kim WJ, Kim J, Jeong CY, Park H, Hong YC, et al. Prenatal Exposure to Traffic-Related Air Pollution and the DNA Methylation in Cord Blood Cells: MOCEH Study. Int J Environ Res Public Health. 2022 Mar 10;19(6):3292.

19. Aguilera J, Han X, Cao S, Balmes J, Lurmann F, Tyner T, et al. Increases in ambient air pollutants during pregnancy are linked to increases in methylation of IL4, IL10, and IFNγ. Clin Epigenetics. 2022 Mar 14;14(1):40.

20. Textor J, van der Zander B, Gilthorpe MS, Liskiewicz M, Ellison GT. Robust causal inference using directed acyclic graphs: the R package “dagitty.” Int J Epidemiol. 2016 Dec 1;45(6):1887–94.
